# Age Differences in the Use/Efficacy of Emotion Coping Strategies among Adults with Chronic Pain: A Scoping Review

**DOI:** 10.1093/geroni/igab046.3223

**Published:** 2021-12-17

**Authors:** Gillian Fennell, Elaine Wethington, M Carrington Reid, Erica Sluys, Kelsey Donovan, Valeria Cardenas, Elizabeth Zelinski, Susan Enguidanos

**Affiliations:** 1 University of Southern California, Los Angeles, California, United States; 2 Cornell University, Ithaca, New York, United States; 3 Weill Cornell Medical College, New York, New York, United States; 4 University of Massachusetts Amherst, Amherst, Massachusetts, United States

## Abstract

Active coping strategies (e.g., exercise and pharmacological treatments) typically do not leave chronic pain patients completely pain-free. Therefore, individuals turn to emotion-focused strategies to cope with associated impairment and psychosocial consequences. General coping strategy use has been shown to differ by age. This scoping review explored age differences in the use and effectiveness of emotion-focused strategies in adults experiencing chronic pain. Studies were located via advanced searches in PubMed, PsycINFO, CINAHL, Embase, Web of Science, and Proquest Dissertations and Theses Global and referral. Two reviewers independently conducted abstract screenings and full-text extractions. Conflicts were discussed and resolved by the PI. We identified 15 studies that met our inclusion criteria, of which 14 met criteria for high methodologic quality. The majority of studies utilized the Coping Strategies Questionnaire to assess differential use of pain-coping strategies. The remaining studies used one of five other questionnaires. Only one study examined the differential effect of age on the efficacy of emotion-focused strategies. Five of the eight studies that examined hoping/prayer coping reported the strategy’s positive association with age. Age was not associated with ignoring pain or reinterpreting pain sensations in any of the eight studies in which these strategies were measured. We concluded that older age was associated with the use of praying/hoping as a means of coping with pain. No other consistent associations between age and other measured coping strategies were identified. Future research should account for auxiliary stressors and pain characteristics while investigating the differential effect of age on pain coping efficacy.

